# The CD14^+^CD16^+^ Inflammatory Monocyte Subset Displays Increased Mitochondrial Activity and Effector Function During Acute *Plasmodium vivax* Malaria

**DOI:** 10.1371/journal.ppat.1004393

**Published:** 2014-09-18

**Authors:** Lis R. V. Antonelli, Fabiana M. S. Leoratti, Pedro A. C. Costa, Bruno C. Rocha, Suelen Q. Diniz, Mauro S. Tada, Dhelio B. Pereira, Andrea Teixeira-Carvalho, Douglas T. Golenbock, Ricardo Gonçalves, Ricardo T. Gazzinelli

**Affiliations:** 1 Laboratório de Immunopatologia, Centro de Pesquisas René Rachou, Fundação Oswaldo Cruz, Belo Horizonte, Minas Gerais, Brazil; 2 Departamento de Bioquímica e Imunologia, Universidade Federal de Minas Gerais, Belo Horizonte, Minas Gerais, Brazil; 3 Centro de Pesquisas em Medicina Tropical de Rondônia, Porto Velho, Rondônia, Brazil; 4 Laboratório de Biomarcadores de Diagnóstico e Monitoração, Centro de Pesquisas René Rachou, Fundação Oswaldo Cruz, Belo Horizonte, Minas Gerais, Brazil; 5 Division of Infectious Diseases and Immunology, University of Massachusetts Medical School, Worcester, Massachusetts, United States of America; 6 Departamento de Patologia Geral, Universidade Federal de Minas Gerais, Belo Horizonte, Minas Gerais, Brazil; McGill University, Canada

## Abstract

Infection with *Plasmodium vivax* results in strong activation of monocytes, which are important components of both the systemic inflammatory response and parasite control. The overall goal of this study was to define the role of monocytes during *P. vivax* malaria. Here, we demonstrate that *P. vivax*–infected patients display significant increase in circulating monocytes, which were defined as CD14^+^CD16^−^ (classical), CD14^+^CD16^+^ (inflammatory), and CD14^lo^CD16^+^ (patrolling) cells. While the classical and inflammatory monocytes were found to be the primary source of pro-inflammatory cytokines, the CD16^+^ cells, in particular the CD14^+^CD16^+^ monocytes, expressed the highest levels of activation markers, which included chemokine receptors and adhesion molecules. Morphologically, CD14^+^ were distinguished from CD14^lo^ monocytes by displaying larger and more active mitochondria. CD14^+^CD16^+^ monocytes were more efficient in phagocytizing *P. vivax*-infected reticulocytes, which induced them to produce high levels of intracellular TNF-α and reactive oxygen species. Importantly, antibodies specific for ICAM-1, PECAM-1 or LFA-1 efficiently blocked the phagocytosis of infected reticulocytes by monocytes. Hence, our results provide key information on the mechanism by which CD14^+^CD16^+^ cells control parasite burden, supporting the hypothesis that they play a role in resistance to *P. vivax* infection.

## Author Summary

Malaria, caused by a protozoa parasite, *Plasmodium*, affects more than 200 million people per year. The infection triggers an acute febrile illness, the paroxysms, occurring every 48 or 72 hours depending on the species. *Plasmodium vivax*, in most cases, does not cause severe malaria, but it is the most geographically widespread parasite responsible for human disease and causes substantial costs to individuals and governments. Once the parasite reaches the blood stream, they infect reticulocytes that can be destroyed by phagocytes. Our goal was to assess the importance of monocyte subsets during malaria. We found that *P. vivax* infection causes an increase in frequency of circulating monocytes, which were defined as classical, inflammatory, and patrolling, based on the expression of membrane molecules. Classical and inflammatory monocytes produced higher levels of pro-inflammatory cytokines and were distinguished from patrolling monocytes by displaying larger and more active mitochondria. Importantly, inflammatory monocytes were more efficient phagocytes; produced high levels of intracellular reactive oxygen species and TNF and consequently control better *Plasmodium vivax* infection. Hence, our results support the hypothesis that CD14^+^CD16^+^ monocytes display effector functions involved in parasite control during malaria.

## Introduction

*Plasmodium vivax* is the most widely distributed malaria parasite and responsible for approximately 70–80 million cases, annually. In addition, *P. vivax* is responsible for the majority of malaria cases and represents a significant impediment to social and economic development in Latin America and Asia [1]. Both innate and acquired immunity are thought to play critical role in host resistance to infection and pathogenesis of malaria [2], [3]. However, the mechanisms by which the innate immune response mediate resistance to *Plasmodium* infection or promote a deleterious systemic inflammation associated with malaria sepsis are poorly understood [2]. This is particularly true in the case of *P. vivax* malaria [4].

The blood is the main tissue affected during *P. vivax* malaria since sequestration is not a central event in this infection. When parasitized reticulocytes rupture in the blood stream, parasite components are sensed by the innate immune receptors and activate monocytes [5]. The innate immune system recognizes *Plasmodium sp.* through different pattern-recognition receptors expressed by monocytes and initiates a broad spectrum of defense mechanisms [6], [7], [8], [9], [10]. Importantly, the same immune mediators involved in host resistance, such as pro-inflammatory cytokines are also thought to mediate pathology during acute malaria episodes [8], [11], [12]. However, the full spectrum of monocyte subsets and the specific functions of each monocyte population during malaria have not been defined.

Besides supplying peripheral tissues with macrophage and dendritic cell (DC) precursors, monocytes contribute directly to immune defense against microbial pathogens [13], [14], [15]. Monocytes were initially identified by their expression of large amounts of CD14 [16], [17]. However, recent studies have revealed that monocytes in human peripheral blood are heterogeneous and can be divided into three distinct subsets described based on their expression of phenotypic markers. These cells are referred to as, classical (CD14^+^CD16^−^ cells), inflammatory or intermediate (CD14^+^CD16^+^) and patrolling (CD14^lo^CD16^+^) monocytes [18], [19]. Given the importance of monocytes as a major source of pro-inflammatory cytokines and potential effector cells during malaria, in this study, we have attempted to define the role of the various monocyte subsets during *P. vivax* infection. To address this question, we phenotypically and functionally characterized the classical, inflammatory, and patrolling monocytes present in the peripheral blood from patients experiencing acute malaria episodes.

We demonstrate that the frequency of circulating monocytes is elevated during acute infection with *P. vivax* and that the classical and inflammatory monocyte subsets are the primary source of pro-inflammatory cytokines. Importantly, we found that CD16^+^ cells, and in particular the CD14^+^CD16^+^ LFA-1^high^ICAM-1^high^PECAM-1^high^ monocytes display augmented effector functions such as phagocytosis and intracellular reactive oxygen species production and are thus likely to be an important cell subset controlling parasitemia, and mediating host resistance to *P. vivax* infection.

## Methods

### Patients

A total of 35 *P. vivax*-infected patients with uncomplicated malaria were enrolled in this study at Centro de Pesquisa de Medicina Tropical de Rondônia (CEPEM) in Porto Velho, Rondônia, a malaria endemic area in the Amazon region of Brazil. According to the World Health Organization, uncomplicated malaria is a symptomatic infection with malaria parasitemia without signs of severity and/or evidence of vital organ dysfunction. Up to 100 ml of peripheral blood was collected immediately after confirmation of *P. vivax* infection by thick blood smear film and again 30–45 days after chemotherapy (n = 35, ranging from 18 to 66 years old [35±9.5]) (Table S1). Additional 36 *P. vivax*-infected patients were enrolled for reticulocyte analysis: percentage: mean: 1.383, SD: 0.655; median: 1.150, IQR: 0.925–1.650; absolute numbers: mean: 51,779, SD: 18,390; median: 48,100, IQR: 38,970–59,520. Patients were treated for 3 days with chloroquine followed by 7 days with primaquine according to the Brazilian Ministry of Health. *P. vivax* infection and parasitological cure were confirmed by thick blood smear film and polymerase chain reaction (PCR) [20]. Identification of the three species of human malaria parasites was done by nested PCR that targets variant sequences in the small subunit rRNA gene. The clinical manifestations of acute malaria were fever, myalgia, chills, arthralgia, nausea, vomiting or diarrhea. Peripheral blood was also collected from 15 healthy donors (HD) ranging from 21 to 56 years old [32±8] living in Porto Velho and negative for *P. vivax* infection.

### Ethics statement

These studies were performed under protocols reviewed and approved by the Ethical Committees on Human Experimentation from Centro de Pesquisa em Medicina Tropical de Rondônia (CEP-CEPEM 095/2009) and Centro de Pesquisas René Rachou, Fundação Oswaldo Cruz (CEP-CPqRR 2004), the National Ethical Committee (CONEP 15652) from Ministry of Health, Brazil, as well as by the Institutional Review Board from the University of Massachusetts Medical school. Only adults, 18 years old, were enrolled in the study. All patients enrolled in this study provided written informed consent.

### Cellular immunophenotyping

Peripheral blood mononuclear cells (PBMC) were prepared from heparinized venous blood of adult volunteers by Ficoll-Hypaque density gradient centrifugation (GE Healthcare Life Sciences). Cells were stained for surface molecules for 15 minutes at room temperature. Subsequently, PBMC were washed with FACS buffer (PBS and 2%FBS), and fixed and permeabilized according to the manufacture's instruction (Cytofix/Cytoperm, BD Biosciences). Cells were then stained for the intracellular antigens. Cells were finally suspended and maintained in 200 µl of PBS 2% paraformaldehyde (Sigma Aldrich). At least 100,000-gated events were acquired for analysis using FACSCan upgraded with a second laser (5 colors) with Cellquest Pro and Rainbow from Cytek or LSR II with Diva (both from BD Biosciences). The antibody panels included the following: anti-CD31 (clone WM59)-FITC, anti-CD11a (LFA-1) (clone HI111)-FITC, anti-HLA-DR (clone LN3)-PE, anti-CD14 (clone 61D3)-APC, anti-CD62L (clone DREG56)-APC, anti-HLA-DR (clone LN3)-efluor 780, anti-CX3CR1 (clone 2A9-1)-biotin, anti-CD106 (VCAM-1) (clone STA)-biotin and streptavidin-PE, all purchased from eBioscience; anti-CD54 (ICAM-1) (clone HA58)-PE, anti-TNF-α (clone MAb11)-PE, anti-IL-6 (clone MQ2-13A5)-PE, anti-CD197 (CCR7) (clone 3D12)-PECy7 and anti-CD16 (clone 3G8)-PercPCy5.5, purchased from BD Biosciences; anti-CCR2 (clone 48607)-PE, purchased from R&D Systems. Data were analyzed using FlowJo Version 9.3.2 (TreeStar). A forward scatter height (FSC-H) and a side scatter height (SSC-H) gate were used to initially remove debris and to capture leucocytes. CD14 versus SSC-H gate was then used to select monocytes. An additional HLA-DR versus CD16 gate was performed to exclude possible contamination by neutrophils (CD16^+^HLA-DR^−^). A more detailed analysis of monocyte subpopulations was done based on CD14 and CD16 expression and here designated as: classical monocytes (CD14^+^CD16^−^), inflammatory monocytes (CD14^+^CD16^+^), and patrolling monocytes (CD14^l^°CD16^+^). The activation/cell presentation molecule, HLA-DR; the cellular adhesion molecules, VCAM-1, ICAM-1, PECAM-1, and LFA-1; the chemokine receptors, CCR2, CX3CR1 and CCR7; and the cytokine TNF-α was analyzed within CD14^+^ cells and also within each monocyte subpopulation. Data is shown in frequencies or mean fluorescence intensity (MFI). In the latter, when graphs are overlaid, the y-axis is left on automatic scaling and the axis represents % of Maximum. This normalization is used because different numbers of events is collected for the monocyte subsets analyzed and allow us to focus on the important and relevant variations between the levels of expression of different markers on the x-axis.

### Cytokine measurements

IL-6, IL-8 and IL-10 were measured in cryopreserved plasma using the Cytometric Bead Array kit (CBA, BD Biosciences Pharmingen) as recommended by the manufacturer. The concentration of cytokines in each sample was calculated using the BD FCAP Array Software v 1.0.1 (BD Biosciences).

### *P. vivax*-parasitized reticulocytes enrichment and staining

The red blood cells pellet from the Ficoll-Hypaque (GE Healthcare Life Sciences) density gradient centrifugation was harvested and washed three times and then resuspended in RPMI 1640 medium (Sigma Aldrich) to a final hematocrit of 10%. Five milliliters of this suspension was overlaid on a 5 mL 45% Percoll solution in a 15 mL tube. After centrifugation, floating mature Pv-reticulocytes (Pv-Ret) were collected and resuspended in RPMI 1640 [21], [22]. The enrichment of Pv-Ret was assessed by optic microscopy and a purity of 95% was obtained. Where indicated, enriched Pv-Ret were stained with 1 µM carboxyfluorescein succinimidyl ester (CFSE) at 1×10^6^ cells/mL for 8 minutes at room temperature before phagocytosis assays (Molecular Probes-Invitrogen). After stained Pv-Ret were washed three times in RPMI and 10%FBS.

### Expression of NADPH subunits by monocyte subsets

P47phox and p67phox expression was assessed by incubating PBMC in suspension at 37°C in complete RPMI 1640 supplemented with 2 µM glutamine, 10 mM HEPES and 50 µM 2-ME for 3 hours in medium alone and with *P. vivax*-Ret (0.5 Pv-Ret/PBMC) in the presence of 10% immune serum. PBMC were then washed with FACS buffer, stained for surface molecules for 15 minutes at room temperature, fixed and permeabilized according to the manufacture's instruction (Cytofix/Cytoperm, BD Biosciences).. Cells were then stained for the intracellular antigens (p47phox and p67phox) for 20 minutes, following additional 20 minutes incubation with anti-IgG1. After staining, plates were kept on ice for 15 minutes and cells were harvested with ice-cold PBS, 2.5 mM EDTA and maintained in 200 µl of PBS 2% paraformaldehyde. At least 100,000-gated events were acquired for analysis using a LSRFortessa (BD Biosciences). The antibody panels included the following: anti-CD16 (clone 3G8)-Alexa Fluor 700, anti-HLA-DR (Tu39)-FITC, anti-CD14 (clone M5E2)-APC, purified anti-phox47 (1/p47Phox) and anti-phox67 (D-6), and anti-IgG1 (A851)-PE. The MFI of each monocyte subpopulation expressing the NADPH subunits was determined by flow cytometry. Data were analyzed using FlowJo Version X 10.0.7.

### Phagocytosis

Phagocytosis was assessed by incubating PBMC in suspension using 96-wells polystyrene plates at 37°C in complete RPMI 1640 supplemented with 10% heat-inactivated FCS, 2 µM glutamine, 10 mM HEPES, and 50 µM 2-ME for 1, 4 and 12 hours with *P. vivax*-Ret previously stained with CFSE (0.5 Pv-Ret/PBMC) in the absence of immune serum and in the presence of serum or inactivated serum. In some experiments monoclonal anti-CD11a (1 mg/mL) (clone G43-25B, BD), anti-CD31 (0.5 mg/mL) (clone WM59), anti-CD36 (0.5 mg/mL) (clone CB38) and anti-CD54 (1 mg/mL) (clone HA58) blocking antibodies were added in the cultures 30 minutes before the addition of *P. vivax*-Ret. After staining, plates were kept on ice for 15 minutes and cells were harvested with ice-cold PBS containing 2.5 mM EDTA. The frequencies of total monocytes and each monocyte subpopulation positive for CFSE were determined by flow cytometry and data were analyzed using FlowJo Version 9.3.2 (TreeStar).

### ROS detection

To detect ROS production at the single cell level, the Image-iT LIVE Green Reactive Oxygen Species Detection kit (Invitrogen) was used following the manufacturer's instructions. Briefly, PBMC were washed twice with PBS and incubated in medium alone or with Pv-Ret (0.5 Pv-Ret/PBMC) or phorbol 12-myristate 13-acetate (PMA, 10 ng/mL) and ionomycin (500 ng/mL). Pre-warmed 25 µM 5-(and-6)-carboxy-2′,7′-dichlorodihydrofluorescein diacetate (carboxy-H_2_DCFDA) was added to the cells for 3 hours at 37°C. Mitochondrial ROS was assessed by MitoSox red mitochondrial superoxide indicator (Invitrogen) following the manufacturer's instructions. Briefly, PBMC were washed twice with HBSS and incubated in medium alone or with Pv-Ret (0.5 Pv-Ret/PBMC) or PMA (10 ng/mL) and ionomycin (500 ng/mL) and 10 µm MitoSox. After 30 minutes incubation, monoclonal antibodies against CD14 and CD16 were added to cell cultures to allow monocyte subpopulations analysis. Cell suspension was washed after additional 30 minutes incubation with HBSS at 37°C. Plates were kept on ice for 15 minutes and cells were harvested with ice-cold PBS containing 2.5 mM EDTA. Cells were acquired by flow cytometry and data were analyzed using FlowJo Version 9.3.2.

### Purification of monocyte subpopulations

After PBMC preparation CD14^+^CD16^−^, CD14^+^CD16^+^ and CD14^lo^CD16^+^ monocytes from HD and *P. vivax*-infected patients were sorted with a FACSAria II cell sorter (BD Biosciences), using the combination of antibodies described above. CD14^+^CD16^−^, CD14^+^CD16^+^ and CD14^l^°CD16^+^ monocytes were then collected and fixed with 2.5% buffered glutaraldehyde solution, 0.1 M, for electron microscopy or with RLT buffer (QIAGEN) supplemented with β-mercaptoethanol for mRNA detection and nanostring analysis as described below.

### Electron microscopy

After FACS-sorting, cells were prepared as previously described [23], [24]. Briefly, cells were fixed in 2.5% buffered glutaraldehyde solution, 0.1 M, pH 7.2, 6 h, 8°C. Cells were then washed with the same buffer. The pellets were included in phosphate buffer, 4% agarose and left overnight at 4°C. Next, the cells were fixed in a mixture of 1% osmium tetroxide and 1.5% (w/v) potassium ferrocyanide, dehydrated in a graded series of ethanol solutions, infiltrated, and embedded in Araldite 502 (Electron Microscopy Sciences, Hatfield, PA, USA). After polymerization, thin sections were obtained using a diamond knife on a Sorvall MT-2B ultramicrotome (Dupont, Wilmington, DE, USA) and mounted on uncoated 200-mesh copper grids (Ted Pella, Inc., Redding, CA, USA). Sections were stained with 2% uranyl acetate and Reynolds lead citrate and then analyzed using transmission electron microscopy (EM 10A Zeiss).

### mRNA detection

mRNA was assessed by nanostring analysis [25]. nCounter CodeSets were constructed for detecting selected human-specific genes. A total of 1×10^4^ cells of each subset were lysed in RLT buffer (QIAGEN) supplemented with β-mercaptoethanol. This lysate was mixed with capture and reporter probes, hybridized to the Codeset for 16 hr and loaded onto the nCounter prep station, and then quantified with the nCounter Digital Analyzer. For side-by-side comparisons of nCounter experiments, data were normalized in two ways described previously [26]. Briefly, the first normalization was for small variations utilizing the internal positive controls that are present in each CodeSet. Then the samples were normalized with 7 housekeeping genes that were included in the CodeSet. The data was analyzed with n Solver software. The heatmap was constructed using log2 transformed data and the Tiger Multi Experiment Viewer software.

### Statistical analysis

Statistical analysis was performed using GraphPad Prism software, version 5.0. The results were analyzed using two-tailed paired t-test. Wilcoxon testing was used when data did not fit a Gaussian distribution. The results were analyzed using unpaired t-test when two groups were compared. Mann-Whitney (MW) test was used when a normality test failed. Analyses were also done between HD (represented in the graphs as dashed line) and patients after cure and no significant differences were found. The correlation analyses were performed using the Spearman's rank. Differences were considered to be statistically significant, when *p*≤0.05.

## Results

### *P. vivax*-infected patients display higher levels of plasma cytokines accompanied by increased frequencies of circulating monocytes

**Figure 1:**
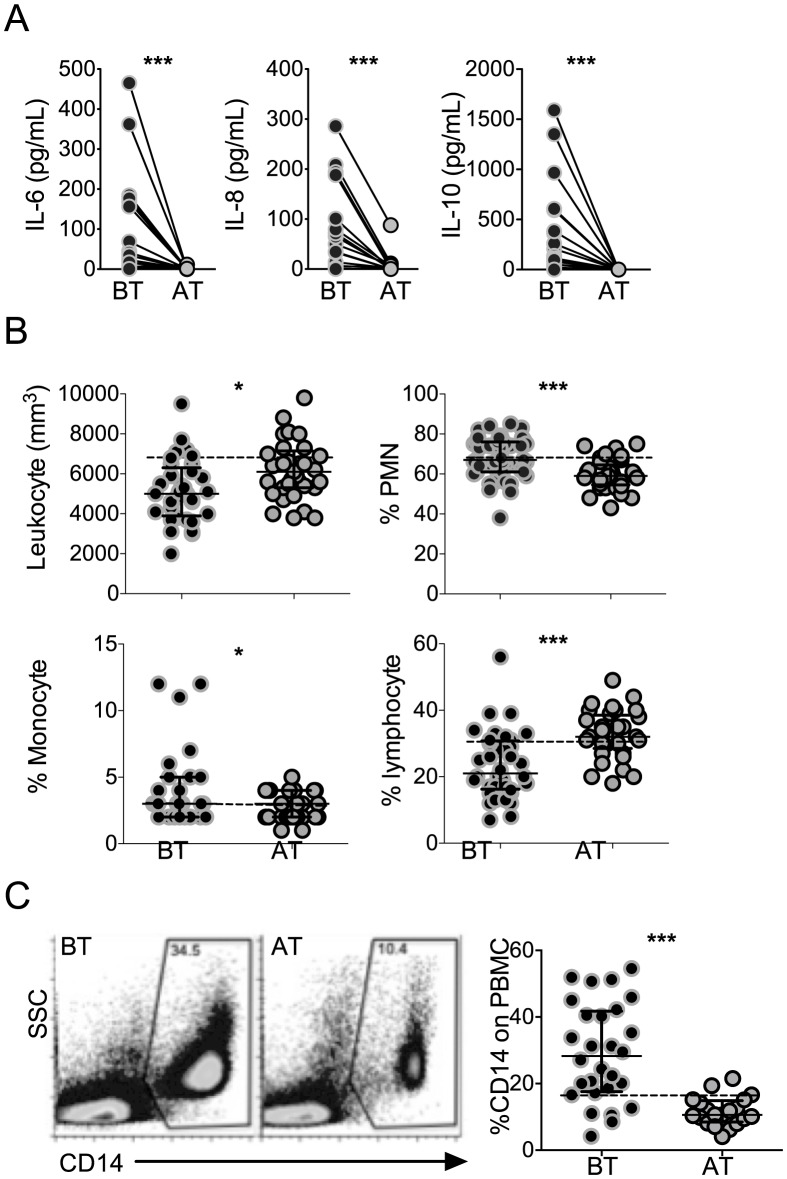
*P. vivax*-infected patients display higher levels of cytokines accompanied by increased frequencies of circulating monocytes. (A) IL-6, IL-8 and IL-10 were measured in plasma of *P. vivax*-infected individuals before (BT, black circles) and 30–45 days after (AT, grey circles) treatment (n = 20). Levels of cytokine were measured by Cytometric Bead Array. (B) Leukocyte counts and frequencies from *P. vivax*-infected individuals before (BT, black circles) and after (AT, grey circles) treatment were assessed at a clinical laboratory (n = 33). C) Representative density plots of CD14^+^ monocytes (left panel) and frequencies of CD14^+^ monocytes (right panel) within PBMCs from *P. vivax*-infected individuals (BT, n = 28 and AT, n = 20). Circles indicate individual patients and lines represent median values and interquartile ranges. Dotted lines represent medians of a given measurements from healthy donors. *p<0.05, ***p<0.01.

High levels of the pro-inflammatory cytokines, IL-6 and IL-8, and regulatory cytokine IL-10, were found in the circulation of *P. vivax*-infected patients before treatment initiation, when compared to the same patients after anti-malarial therapy (Figure 1A). While the cytokinemia of *P. vivax*-infected patients was significantly higher than individuals after cure, the absolute numbers of total leukocytes decreased with infection (Figure 1B). The frequencies of lymphocytes were lower, whereas the proportions of polymorphonuclear cells and monocytes were higher in symptomatic malaria patients. Monocyte frequencies were also assessed within PBMC by flow cytometry. The frequencies of CD14^+^ monocytes were significantly higher in *P. vivax*-infected patients than in the same individuals after treatment (Figure 1C).

### *P. vivax* infection alters the expression of HLA-DR, adhesion molecules and chemokine receptors on circulating monocytes

**Figure 2:**
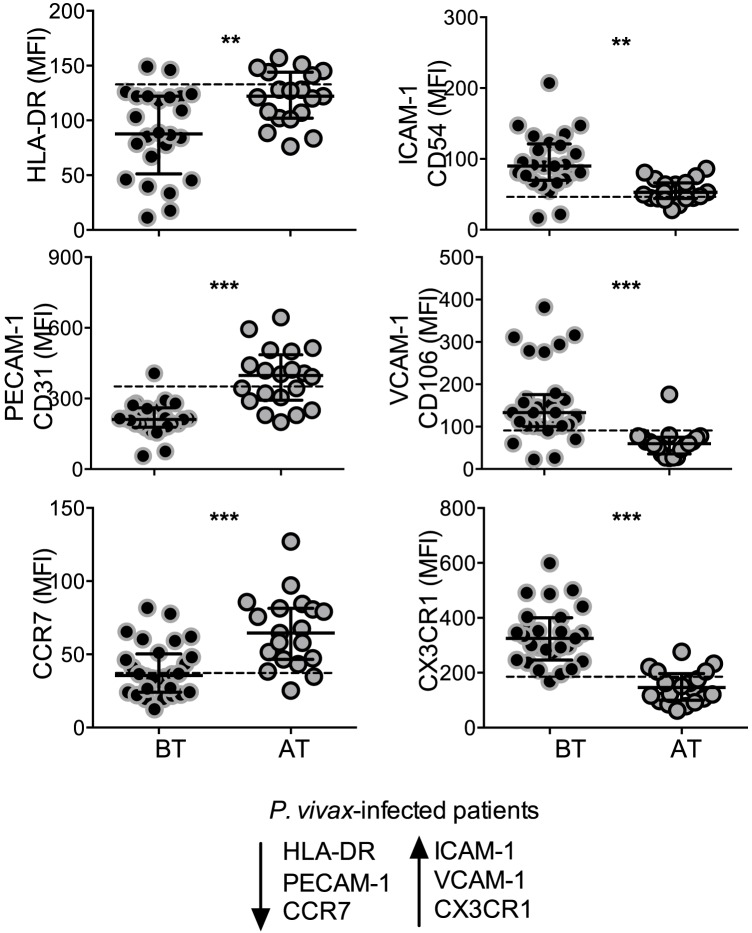
*P. vivax* infection alters the expression of activation markers, adhesion molecules and chemokine receptors on circulating monocytes. Mean fluorescence intensity (MFI) of HLA-DR (BT, n = 24 and AT, n = 19), CD31 (BT, n = 25 and AT, n = 20), CCR7 (BT, n = 28 and AT, n = 19) (left panels, from the top to the bottom), CD54 (BT, n = 25 and AT, n = 19), CD106 (BT, n = 28 and AT, n = 19), CX3CR1 (BT, n = 28 and AT, n = 20) (right panels, from the top to the bottom) was evaluated on monocytes from *P. vivax*-infected subjects, before (BT, black circles) and 30–45 days after treatment (AT, grey circles). Circles indicate individual patients and lines represent median values and interquartile ranges. Dotted lines represent medians of a given measurements from healthy donors. Levels of the molecules above were measured by flow cytometry. **0.05>*p*>0.01, *** *p*<0.01.

The expression of the activation marker, HLA-DR, cell adhesion molecules, CD54, CD106, CD31, and chemokine receptors, CXCR3, CCR7, was analyzed on circulating monocytes (Figure 2). Significantly lower levels of HLA-DR were found on monocytes from *P. vivax*-infected patients when compared to the same patients after treatment (Figure 2). Monocytes from acute malaria patients also displayed significantly lower levels of the adhesion molecule CD31 and the chemokine receptor CCR7 (Figure 2). In contrast, significantly higher expression of the adhesion molecules CD106 (VCAM-1), CD54 (ICAM-1), and the chemokine receptor CX3CR1, were observed on monocytes from acute malaria patients before treatment initiation (Figure 2). The expression of all these molecules on monocytes from malaria patients reached the levels found in healthy donors when analyzed 30 days after treatment. Thus, monocytes from *P. vivax*-infected patients exhibit a distinct activation state during acute infection.

### Monocyte subpopulations from *P. vivax*-infected patients display distinct activated phenotypes

**Figure 3:**
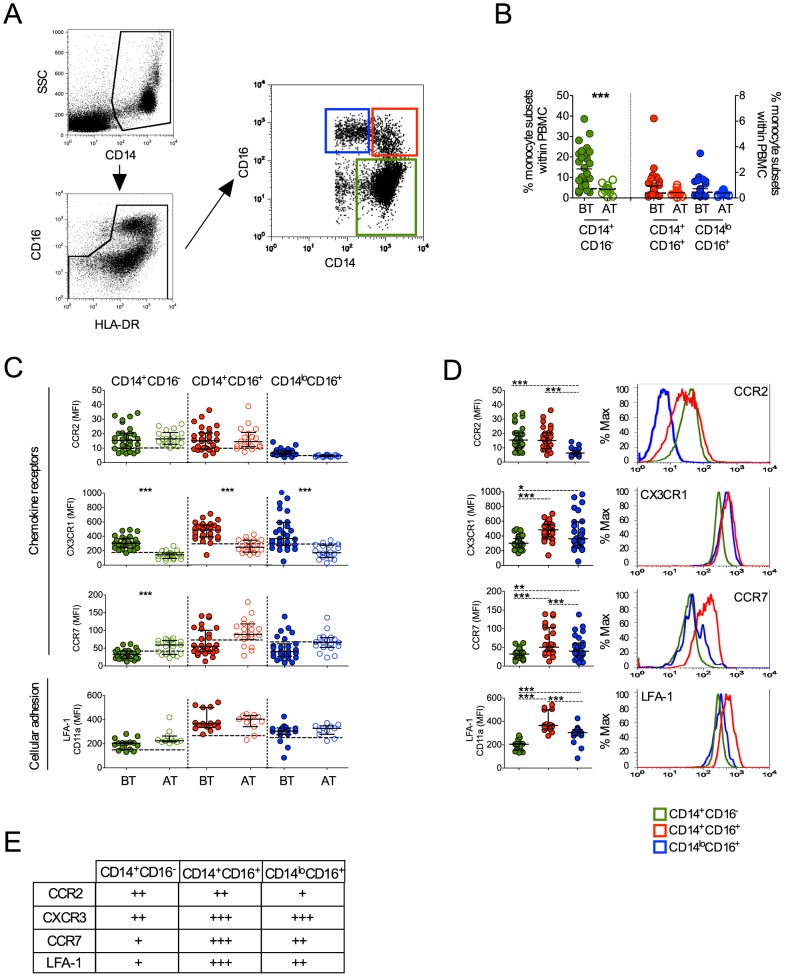
Characterization of monocyte subsets in malaria patients. (A) Representative dot plots showing the gate strategy for the identification of CD14^+^CD16^−^ (green gate), CD14^+^CD16^+^ (red gate), CD14^lo^CD16^+^ (blue gate) monocyte subsets. CD14^+^CD16^−^, CD14^+^CD16^+^ and CD14^lo^CD16^+^ monocytes are represented by green, red and blue symbols. (B) Frequencies of CD14^+^CD16^−^, CD14^+^CD16^+^ and CD14^lo^ monocytes within PBMC from *P. vivax*-infected patients before (BT, filled symbols) and 30–45 days after treatment (AT, open symbols) (n = 28). (C) Mean fluorescence intensity (MFI) of CCR2 (BT, n = 28 and AT, n = 18), CX3CR1 (BT, n = 26 and AT, n = 19), CCR7 (BT, n = 28 and AT, n = 19) and LFA-1 (BT, n = 15 and AT, n = 11) (from the top to the bottom) was evaluated on monocyte subsets (CD14^+^CD16^−^ (left panel), CD14^+^CD16^+^ (middle panel), CD14^lo^CD16^+^ (right panel)) from *P. vivax*-infected subjects, before and 30–45 days after treatment. Dotted lines represent medians of a given measurements from healthy donors. (D) Scattered dot plots (left panels) and representative histograms (right panels) showing MFI of the molecules described above on CD14^+^CD16^−^, CD14^+^CD16^+^ and CD14^lo^CD16^+^ monocytes from *P. vivax*-infected patients before treatment (open histograms). Levels of the molecules above were measured by flow cytometry. Circles indicate individual patients and lines represent median values and interquartile ranges. (E) Levels of molecules expressed by the monocyte subsets analyzed according to D. * *p*<0.05, **0.05>*p*>0.01, ***p<0.01.

As noted above, human monocyte subsets can be distinguished by flow cytometry based on the expression of CD14 and CD16 [18]. The majority of monocytes express CD14 but not CD16, and those expressing CD16 can be subdivided into two subpopulations, CD14^+^CD16^+^ and CD14^lo^CD16^+^ cells (Figure 3A). They are also slightly different in granularity as previously described and shown in Figure S1 [18]. To exclude any contaminating neutrophils, only HLA-DR^+^ cells were included in the analysis and CD16^+^ cells were included only if they also expressed HLA-DR. Higher frequencies of CD14^+^CD16^−^ monocytes were found in *P. vivax*-infected patients when compared with patients after treatment (Figure 3B). The frequencies of CD14^+^CD16^+^ and CD14^lo^CD16^+^ monocytes did not differ significantly between malaria patients before and after treatment.

Chemokine receptors and the adhesion molecule, LFA-1, have been reported to be differently expressed on monocyte subsets. Previous studies described that CCR2 is expressed by both CD14^+^CD16^−^ and CD14^+^CD16^+^ but not by CD14^lo^CD16^+^ monocytes [18], [27]. Similarly in *P. vivax*-infected patients, low levels of CCR2 were found in CD14^+^CD16^−^ and CD14^+^CD16^+^ monocytes and, as expected, CCR2 was barely expressed by CD14^lo^CD16^+^ cells (Figure 3C and 3D). In addition, no changes in LFA-1 expression in the different monocyte subsets were observed in *P. vivax* patients as a result of treatment (Figure 3C). Higher LFA-1 levels were observed in CD14^+^CD16^+^, followed by CD14^lo^CD16^+^ and CD14^+^CD16^−^ monocytes (Figure 3D). The expression of CX3CR1 along with LFA-1 has been implicated in the ability of CD14^lo^CD16^+^ monocytes to crawl on the inner surface endothelium of blood vessels [18], [28]. Importantly, *P. vivax* infection triggered a significantly increased expression of CX3CR1 on CD14^+^CD16^−^, CD14^+^CD16^+^ and CD14^lo^CD16^+^ monocytes (Figure 3C). CX3CR1 was expressed at lower levels by CD14^+^CD16^−^ monocytes, when compared to the CD16^+^ populations (Figure 3D). Lastly, changes in CCR7 expression were observed only on CD14^+^CD16^−^ monocytes, with those from *P. vivax* infected patients expressing lower levels of CCR7 compared to monocytes from treated individuals (Figure 3C). Thus, all the monocyte subpopulations from *P. vivax*-infected display a distinct phenotypic profile in patients undergoing acute malaria compared with the same individuals after treatment.

### *P. vivax* induces a variety of inflammatory genes during malaria infection

**Figure 4:**
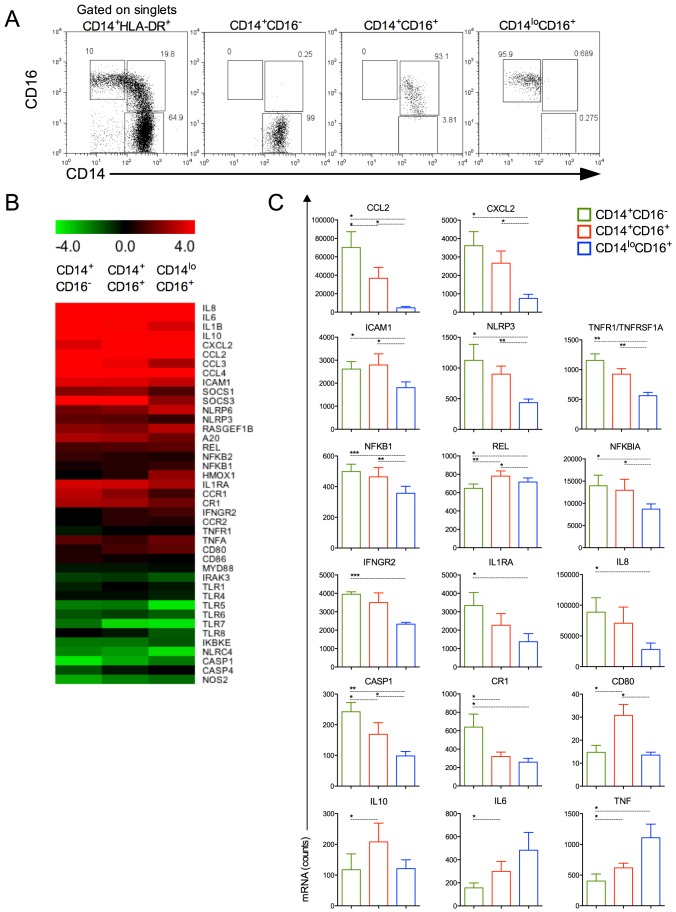
Monocyte subsets display distinct gene expression. CD14^+^CD16^−^, CD14^+^CD16^+^ and CD14^lo^CD16^+^ monocytes from healthy donors and *P. vivax*-infected patients were isolated by FACS. (A) Representative dot plots showing monocyte subpopulations before (left panel) and after FACS-sorting (right panels). (B) A nanostring analysis of monocyte subsets from 5 patients infected with *P. vivax* and 5 healthy donors. Heatmap representation of 41 differentially regulated genes upon malaria infection compared to healthy donors is depicted. (C) *CCL2*, *CXCL2*, *ICAM1*, *NLRP3*, *TNFR1/TNFRSF1A*, *NFKB1*, *REL*, *NFKB1A*, *IFNGR2*, *IL1RA*, *IL8*, *CASP1*, *CR1*, *CD80*, *IL10*, *IL6* and *TNF* were differentially induced among CD14^+^CD16^−^ (green bars), CD14^+^CD16^+^ (red bars) and CD14^lo^CD16^+^ (blue bars) monocytes from *P. vivax*-infected patients. *p<0.05, **0.05>*p*>0.01, ***p<0.01.

We next FACS-sorted the CD14^+^CD16^−^, CD14^+^CD16^+^ and CD14^lo^CD16^+^ monocyte subpopulations from healthy donors and *P. vivax*-infected patients (Figure 4A). The purity of each cell population is shown in Figure 4A. Since the numbers of each circulating monocyte subset obtained from the FACS-sort was limited, we chose to assess the expression of several genes involved in inflammatory responses by nanostring [25]. The expression of 72 selected genes involved in innate immune response, cell adhesion, migration and phagocytosis was evaluated, and we found that 41 genes had their expression significantly altered in at least one of the monocyte subpopulations upon *P. vivax* infection (Figure 4B). Differences greater than 4-fold cannot be appreciated in the heatmap, once the range from −4.0 to +4.0 fold was selected to better reveal differences in the majority of the genes induced by malarial infection in monocyte subsets. Once changes in gene expression were detected upon *P. vivax* infection, the expression of each of these genes was compared among the monocytes subset from *P. vivax*-infected patients (Figure 4C). In general, the CD14^+^ subpopulations, *i.e.*, the classical and inflammatory monocytes, expressed higher levels of RNAs encoded by pro-inflammatory genes. The chemokines CCL2 and CXCL2 were highly expressed by CD14^+^CD16^−^ and CD14^+^CD16^+^ subpopulations, but were expressed at lower levels by CD14^lo^CD16^+^ monocytes (Figure 4C). The same pattern of expression was observed for TNFR1/TNFRSF1A and ICAM-1 with CD14^+^CD16^−^ and CD14^+^CD16^+^ expressing higher levels than CD14^lo^CD16^+^ monocytes (Figure 4C). The classical monocytes, CD14^+^CD16^−^ expressed higher counts of mRNA for the receptor for IFN-gamma and for the IL-1 receptor agonist IL-1RA (Figure 4C). The expression of cytokine genes also varied among monocyte subsets. Both *IL6* and *IL10* had higher expression in CD14^+^CD16^+^ compared to CD14^+^CD16^−^ cells. CD14^lo^CD16^+^ monocytes expressed higher levels of mRNA for TNF and lower levels of *IL8* than the CD14^+^CD16^−^ subset. CD14^+^CD16^−^ as well as CD14^+^CD16^+^ monocytes expressed higher amounts of *NLRP3* and *CASPASE1* than patrolling monocytes. The same pattern of expression was observed for NFKB1 and NFKB1A, involved respectively with induction and regulation of cytokine expression, with CD14^+^CD16^−^ and CD14^+^CD16^+^ expressing higher levels than CD14^lo^CD16^+^ monocytes. REL that encodes a protein that is a member of the Rel/NFKB family was more highly expressed on CD14^+^CD16^+^ monocytes than on the other monocyte subpopulations. The expression of the costimulatory molecule CD80 was higher in CD14^+^CD16^+^ than classical and patrolling monocytes (Figure 4C). Together these data indicate that CD14^+^CD16^−^ and CD14^+^CD16^+^ monocytes have a more activated and inflammatory profile than patrolling monocytes during malaria.

### Monocytes from *P. vivax* infected patients exhibit morphologic changes

**Figure 5:**
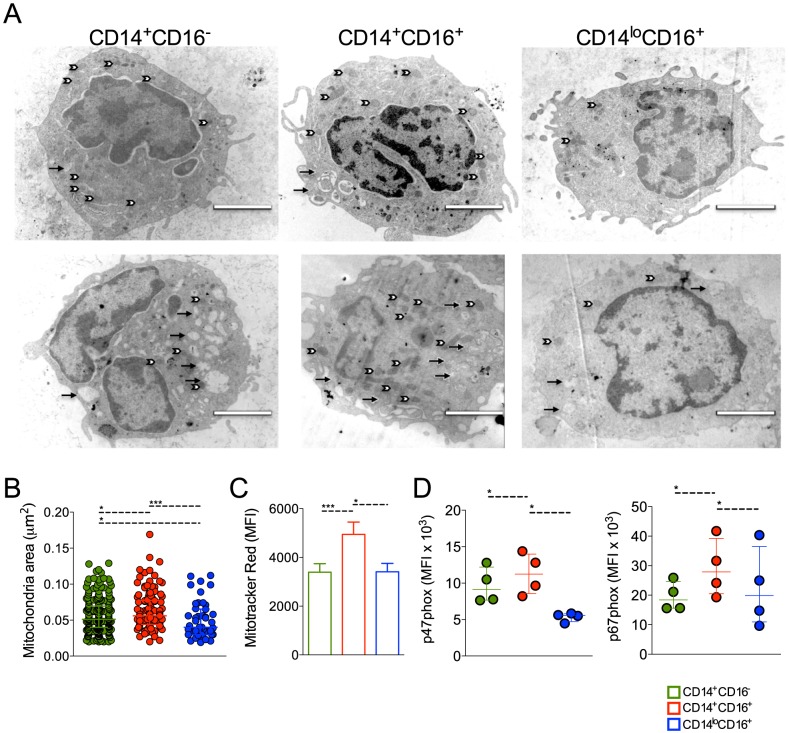
Circulating monocyte subpopulations display distinct morphology, mitochondrial and NADPH subunit content. FACS-sorted CD14^+^CD16^−^, CD14^+^CD16^+^ and CD14^lo^CD16^+^ monocytes from healthy donors and *P. vivax*-infected patients were fixed and prepared for electron microscopy. Monocyte subsets from a single healthy donor (A, upper panel) and a single patient (A, lower panel) shown are representative of the analysis of at least six cells of each monocyte subpopulation per patient and of the analysis of 5 controls and 6 patients. Mitochondria (white arrows) and vesicles (black arrows). Scale bar, 2 µm. (B) Mitochondria area from CD14^+^CD16^−^ (green circles), CD14^+^CD16^+^ (red circles) and CD14^lo^CD16^+^ (blue circles) monocytes was assessed using ImageJ. Circles indicate individual mitochondria. (C) Mitochondria content in CD14^+^CD16^−^ (green bars), CD14^+^CD16^+^ (red bars) and CD14^lo^CD16^+^ (blue bars) monocytes from *P. vivax*-infected patients was measured based on Mitotracker reactivity (n = 11). (D) Mean fluorescence intensity (MFI) of p47phox and p67phox within CD14^+^CD16^−^, CD14^+^CD16^+^, CD14^lo^CD16^+^ monocytes from *P. vivax*-infected patients was measured by flow cytometry 3 hours after culture in medium alone or *P. vivax*-infected reticulocytes (n = 4). Results are representative of 2 independent experiments. Symbols represent individual subject. *p<0.05, ***p<0.01.

Patrolling monocytes can be distinguished from the classical and inflammatory subsets based on size and granularity [18], but no ultrastructural analysis had been previously performed on these cell subpopulations. Electronic microscopy was performed attempting to reveal morphological changes suggestive of functional alterations. FACS-sorted CD14^+^CD16^−^, CD14^+^CD16^+^ and CD14^lo^CD16^+^ monocytes from healthy donors (n = 5) and *P. vivax*-infected patients (n = 6) (Figure 4A) were fixed and processed for ultrastructural analysis by electron microscopy (Figure 5A). CD14^+^CD16^−^ and CD14^+^CD16^+^ monocytes from HD (Figure 5A, upper panel) and *P. vivax*-infected patients (Figure 5A, lower panel) had a larger and a higher number of mitochondria (white arrows) when compared to CD14^lo^CD16^+^ monocytes. All monocyte subsets from *P. vivax*-infected patients displayed morphological features compatible with activation (Figure 5A). Moreover, mitochondria from CD14^+^CD16^+^ cells from *P. vivax*-infected patients were significantly larger than those in the two other monocyte subsets, when mitochondria area was measured using the software ImageJ 1.47K (NIH) (Figure 5B). Mitochondria area was assessed in at least six cells of each monocyte subpopulation per patient.

ROS are generated in multiple compartments and by multiple enzymes in the cell and important contributions include proteins within the plasma membrane, e.g., NADPH oxidases, and mitochondria [29], [30]. Since mitochondria are at least in part responsible for the generation of ROS [31], [32], we further assessed the content of mitochondria in the monocyte subsets from *P. vivax*-infected patients. MitoTracker Red CMX-Ros was used for this propose. The MFI of MitoTracker Red, probe sensitive to membrane potential, was assessed in CD14^+^CD16^−^, CD14^+^CD16^+^ and CD14^lo^CD16^+^ monocyte subsets by flow cytometry (Figure 5C). CD14^+^CD16^−^ and CD14^lo^CD16^+^ monocytes similarly react with Mitotracker Red while significantly higher reactivity was found in CD14^+^CD16^+^ monocytes (Figure 5C). Our data show that CD14^+^CD16^+^ monocytes have larger and more active mitochondria suggesting differential metabolic activity during *P. vivax* infection. The expression of p47phox and p67phox, cytosolic components of the NADPH oxidase, was also measured in monocyte subsets from malaria patients after a short-term culture with *P. vivax*-infected reticulocytes. Higher expression of p47phox and p67phox were found in CD14^+^CD16^+^ monocytes when comparing to their other counterparts (Figure 5D).

### Monocyte subpopulations from malaria patients display distinct levels of molecules involved in cell activation and migration

**Figure 6:**
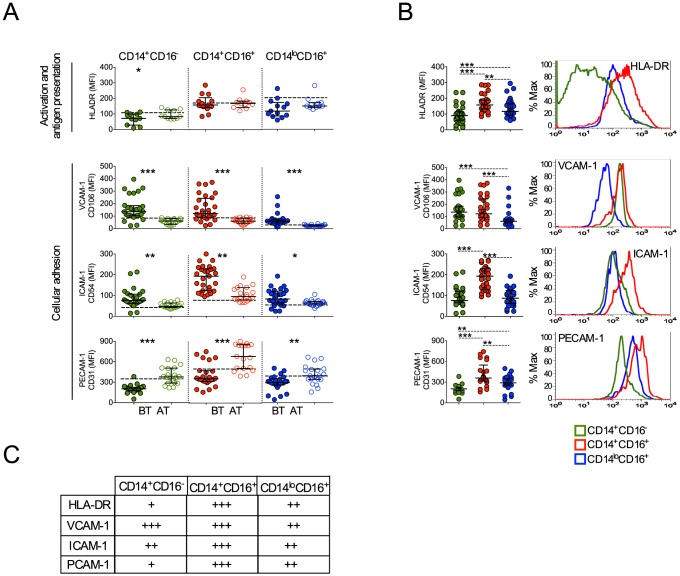
Monocyte subsets from *P. vivax*-infected patients display a highly activated phenotype. CD14^+^CD16^−^, CD14^+^CD16^+^ and CD14^lo^CD16^+^ monocytes are represented by green, red and blue symbols. (A) Mean fluorescence intensity (MFI) of HLA-DR (BT, n = 11 and AT, n = 11), CD106 (BT, n = 26 and AT, n = 19), CD54 (BT, n = 28 and AT, n = 19) and CD31 (BT, n = 25 and AT, n = 20) (from the top to the bottom) was evaluated on monocyte subsets (CD14^+^CD16^−^ (left panel), CD14^+^CD16^+^ (middle panel), CD14^lo^CD16^+^ (right panel)) from *P. vivax*-infected subjects, before (filled symbols) and 30–45 days after treatment (open symbols). Dotted lines represent medians of given measurements from healthy donors. (B) Scattered dot plots (left panels) and representative histograms (right panels) showing MFI of the molecules described above on CD14^+^CD16^−^, CD14^+^CD16^+^ and CD14^lo^CD16^+^ monocytes from *P. vivax*-infected patients before treatment. Levels of the molecules above were measured by flow cytometry. Circles indicate individual patients and lines represent median values and interquartile ranges. (C) Levels of molecules expressed by the monocyte subsets analyzed according to B. *p<0.05, **0.05>*p*>0.01, ***p<0.01.

Upon activation monocytes undergo several changes, including expression of molecules involved with antigen presentation, cell adhesion and migration [33], [34], [35]. CD14^+^CD16^−^ monocytes from patients undergoing *P. vivax* infection expressed lower levels of HLA-DR (Figure 6A). In contrast, the expression of HLA-DR did not differ in CD14^+^CD16^+^ and CD14^lo^CD16^+^ monocytes when cells from malaria patients were compared before and after treatment (Figure 6A). Higher levels of HLA-DR were found in CD14^+^CD16^+^ monocytes compared to their other counterparts during *P. vivax* infection (Figure 6A, B, C).

We also observed that *P. vivax* infection triggered the expression of VCAM-1 and ICAM-1 in all three monocyte subpopulations (Figure 6). In contrast, a decreased expression of PECAM-1 was observed in patients experiencing malaria when compared to the same patients after treatment (Figure 6A). Importantly, CD14^+^CD16^+^ monocytes from *P. vivax*-infected patients expressed the highest levels of ICAM-1 and PECAM-1, when compared to CD14^+^CD16^−^ and CD14^lo^CD16^+^ monocytes (Figure 6B, C). Taken together these results further corroborate that CD14^+^ monocytes, especially the CD14^+^CD16^+^ subset, are highly activated during *P. vivax* malaria.

### CD16^+^CD14^+^ monocytes display elevated phagocytosis

**Figure 7:**
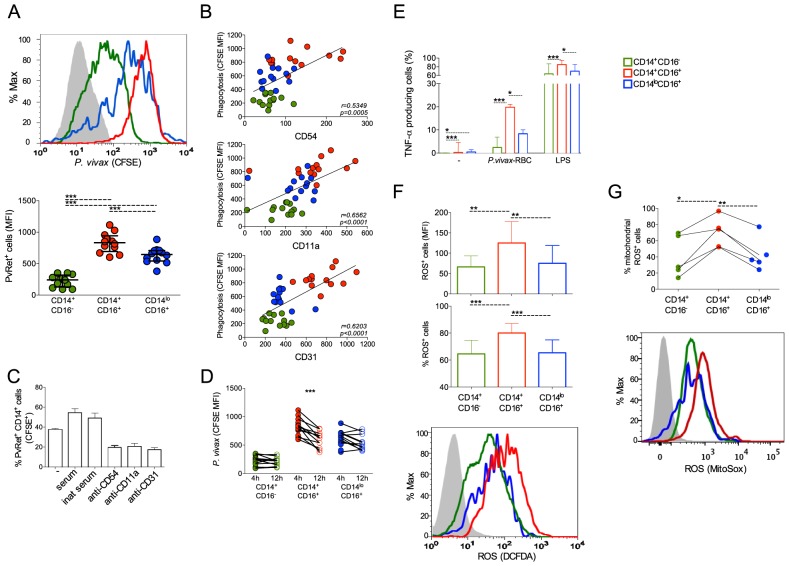
CD16^+^CD14^+^ monocytes display pronounced phagocytic ability than other subsets. CD14^+^CD16^−^, CD14^+^CD16^+^ and CD14^lo^CD16^+^ monocytes are represented by green, red, and blue symbols, respectively. *P. vivax*-infected reticulocytes (Pv-Ret) were purified, CFSE labeled (A, B, C) or not (D, E) and cultured with PBMC from acutely *P. vivax*-infected subjects. (A) Mean fluorescence intensity (MFI) of CFSE within CD14^+^CD16^−^, CD14^+^CD16^+^, CD14^lo^CD16^+^ monocytes exposed to *P. vivax* was measured by flow cytometry after 4 hours of culture. Each circle represents a single patient (n = 12) and lines represent median values and interquartile ranges (B) Correlation between *P. vivax* phagocytosis (CFSE MFI) and CD54, CD11a or CD31 expression by CD14^+^CD16^−^, CD14^+^CD16^+^, CD14^lo^CD16^+^ monocytes. Circles indicate individual patients (n = 13). (C) Phagocytosis of Pv-Ret by CD14+ cells was measured in the absence of serum, presence of inactivated serum and presence of serum, and in the presence of anti-CD54, CD11a and CD31 blocking antibodies (lower graph). Bars represent median values and interquartile ranges (n = 6). (D) MFI of CFSE within CD14^+^CD16^−^, CD14^+^CD16^+^, CD14^lo^CD16^+^ monocytes exposed to *P. vivax* was measured by flow cytometry after 4 and 12 hours of culture. Connecting circles represent values of CFSE MFI of a single patient after 4 and 12 hours of culture, (n = 12) (E) Frequencies of TNF-α producing monocyte subsets were measured after culture with medium alone, Pv-Ret or LPS. Bars represent median values and interquartile ranges (n = 6). (F) ROS production was detected by measuring MFI (top panel) and the proportions (middle panel) of carboxy-H_2_DCDA^+^ CD14^+^CD16^−^, CD14^+^CD16^+^ and CD14^lo^CD16^+^ monocytes after 3 hours incubation with *P. vivax*-infected reticulocytes. Bars represent median values and interquartile ranges (n = 9). Representative histograms showing MFI of carboxy-H_2_DCFDA expressing CD14^+^CD16^−^, CD14^+^CD16^+^, CD14^lo^CD16^+^ monocytes (bottom panel). Grey histogram represents CD14^+^ cells cultured in the absence of Pv-Ret. (G) Mitochondrial ROS was assessed in monocyte subsets from *P. vivax*-infected patients measuring MitoSox by flow cytomery after culture with *P. vivax*-infected reticulocytes (n = 4). Connecting circles represent frequencies of different monocyte subsets producing mitochondrial ROS. Symbols represent individual subject. Representative histograms showing MFI of MitoSox expressing CD14^+^CD16^−^, CD14^+^CD16^+^, CD14^lo^CD16^+^ monocytes (bottom panel). Grey histogram represents MitoSox- cells. *p<0.05, **0.05>*p*>0.01, ***p<0.01.

We used CFSE labeled *P. vivax*-infected reticulocytes (Pv-Ret) to quantify phagocytosis by different monocyte subpopulations from malaria patients before treatment initiation. CD14^+^CD16^+^ cells displayed significantly higher levels of phagocytosis of Pv-Ret than the other monocyte subsets (Figure 7A). The phagocytic ability of CD14^+^CD16^+^ cells was followed by the CD14^lo^CD16^+^ patrolling monocytes, which was significantly better than the CD14^+^CD16^−^ monocytes (Figure 7A). It is important to note that significant differences are found in the phagocytic ability of monocyte subsets when uninfected reticulocytes are purified from healthy donors and co-cultured with PBMC from patients and healthy donors. Much lower frequencies of reticulocytes containing monocytes are detected when cultures are performed with uninfected reticulocytes compared to *P. vivax*-infected reticulocytes (CD14+CD16^−^: 3.54%±0.52% vs. 71.14%±11.11%, CD14^+^CD16^+^: 23.20%±2.18% vs. 98.33%±1.40%, CD14^lo^CD16^+^: 23.18%±4.22% vs. 87.13%±8.10%). The same phenomenon was shown for *P. falciparum* [36].

Interestingly, higher levels of phagocytosis of Pv-Ret correlated with higher expression of the adhesion molecules ICAM-1 (CD54), LFA-1 (CD11a) and PECAM-1 (CD31) by monocytes (Figure 7B). As adhesion molecules, such as CD54, expressed by cell lines interact with *P. vivax*-infected reticulocytes [22], we used blocking antibodies to assess whether phagocytosis of *P. vivax*-infected erythrocytes was dependent on those interactions. Indeed, the phagocytosis of Pv-Ret by CD14^+^ cells was partially blocked by either anti-CD54 or -CD11a or -CD31, as observed by the significantly decreased frequencies of monocytes containing Pv-Ret (Figure 7C). To assess the ability of each monocyte subpopulation to kill Pv-Ret, PBMC were left in cultures for 12 h in the presence of CFSE-labeled-Pv-Ret, and the mean fluorescence intensity of CFSE were measured in each monocyte subset. The MFI of CFSE was only decreased in CD14^+^CD16^+^ monocytes when analyzed 12 h and compared to 4 h of culture (Figure 7D). Both CD14^+^CD16^−^ and CD14^l^°CD16^+^ displayed similar levels of Pv-Ret containing monocytes when the MFI was compare between 4 and 12 h of culture. Important to mention that no significant increase in apoptosis or changes in the proportions of monocyte subsets were detected when PBMC were cultured for 4 and 12 hours in medium or Pv-Ret (Figure S2) (Text S1).

### Higher levels of intracellular TNF and ROS are found in CD14^+^CD16^+^ monocytes compared to their other counterparts

To examine the mechanism of killing, we evaluated the ability of the different monocytes to produce TNF-α and ROS, which are key effector molecules made by activated monocyte/macrophages. Significantly higher frequencies of TNF-α producing cells were found among CD14^+^CD16^+^ monocytes when PBMC were cultured with Pv-Ret (or LPS) (Figure 7E).

ROS production by PBMC and monocyte subsets from *P. vivax*-infected patients was measured by luminescence (RLU) of luminol or fluorescence (RFU) of H_2_DCFDA (Figure S4). PBMC from *P. vivax*-infected patients produced detectable amounts of total ROS spontaneously or in response to PMA, when measured by luminol (Figure S4A) (Text S1). PBMC from acutely infected patients produce higher levels of ROS than the same patients after treatment. In addition, total ROS production was measured in purified monocyte subsets, but no significant differences were found among them (Figure S4B). Intracellular ROS production by each monocyte subpopulation was also measured in the single-cell level by flow cytometry. Consistent with the phagocytosis and TNF-α results, CD14^+^CD16^+^ monocytes exposed to Pv-Ret generated significantly higher levels of ROS than the other monocyte subpopulations (Figure 7F).

We then performed experiments using rotenone and DPI (diphenylene iodonium) to block respectively the mitochondria complex I and NADPH oxidase plus nitric oxide synthases [37], [38], [39]. The inhibition of ROS production was assessed in PBMC by luminescence using luminol and in monocyte subsets using H_2_DCFDA by flow cytomety (Figure S4C and S4D). Rotenone was able to partially inhibit ROS production by PBMC (Figure S4C) and by CD14^+^CD16^+^ cells cultured with parasitized reticulocytes (Figure S4D). When DPI was added in the culture, it was able to abrogated ROS production by PBMC (Figure S4C). Partial inhibition was observed when H_2_DCFDA^+^CD14^+^CD16^+^ cells from malaria patients were cultured with Pv-Ret and assessed by flow cytometry (Figure S4D). To more specifically analyze whether mitochondrial ROS were differentially produced by monocyte subsets during malaria, MitoSox staining was performed. Corroborating with total intracellular ROS production assessed with H_2_DCFDA, CD14^+^CD16^+^ cells were the monocyte subset that most reacted with MitoSox (Figure 7G).

Taken together, our data indicate that CD14^+^CD16^+^ inflammatory monocytes play important effector activity during *P. vivax* infection.

## Discussion

Although pro-inflammatory cytokines play an important role in host resistance to *Plasmodium* infection, various studies reported that they may contribute to deleterious effects during malaria [40], [41], [42]. Thus, the pathways involved on induction of these mediators during malaria, represent checkpoints for immunological intervention to prevent poor outcome of disease. Monocytes have been described as a major source of cytokines during *P. vivax* infection [5]. Although infection with *Plasmodium spp* is known to dramatically alter monocyte differentiation [43], the role of monocyte subsets in host resistance to infection and pathogenesis of malaria remains poorly understood. The findings reported here clearly demonstrate that both the classical (CD14^+^CD16^−^) and intermediate or inflammatory (CD14^+^CD16^+^) monocytes are important sources of cytokines during acute *P. vivax* infection. Intriguingly, the CD14^+^CD16^+^ cells displayed the highest mitochondria content and activity, being an important source of ROS and were the most efficient phagocytes of *P. vivax* infected reticulocytes.

Both in mice and human, different monocyte subsets seem to reflect developmental stages with distinct physiological roles, such as recruitment to inflammatory lesions or entry to normal tissues [17]. Consistent with our results assessing the monocyte subsets in *P. vivax*-infected patients, the majority of monocytes found in steady state, known as classical monocytes, express CD14 but not CD16, and the remaining monocytes express both CD14 and CD16: CD14^+^CD16^+^ (inflammatory) and CD14^lo^CD16^+^ (patrolling) monocytes [18]. This classification still gives rise to discussion. Ziegler-Heitbrock and coworkers has defined CD14^lo^CD16^+^ as non-classical and CD14^+^CD16^+^ as intermediate monocytes [44]. Moreover, some studies have been analyzed monocytes based on the expression of molecules related to differentiation/activation found in macrophages [45], [46]. Despite several of these molecules were assessed in this study many others, such as CD68, CD163 and CD206 have been strongly correlated with different monocyte subsets [45].

As described here, both classical and inflammatory monocytes expressed the chemokine receptor CCR2. It was previously shown in the *P. chabaudi* rodent model of malaria that inflammatory monocytes migrate to spleens, in CCR2 dependent manner, where they are important effector cells implicated in the control of parasite burden, likely through their phagocytic activity and release of ROS [47]. CCR2, the chemokine receptor for CCL2 (also known as monocyte chemotactic protein-1) is a marker for inflammatory monocytes. These monocyte subsets were also shown to express higher levels of mRNA encoding CCL2 when compared to the patrolling monocytes. Similarly, a previous study reported that patients infected with either *P. vivax* or *P. falciparum* have high levels of circulating CCL2 [48]. Classical and inflammatory monocytes from *P. vivax*-infected patients also expressed high levels of inflammatory mediators, including CXCL2 and the receptors for TNF-α, IFN-γ and IL-1. Despite the similarities described above, CD14^+^CD16^+^ monocytes displayed the highest frequencies of TNF-α producing cells when exposed to Pv-Ret. Interestingly, CD14^+^CD16^+^ monocytes also expressed higher mRNA for IL-10 than the other monocyte subsets. It has been described that highly activated effector cells can acquire regulatory features. During *Leishmania* [49], [50], *T. cruzi* [51] and *T. gondii* [52] infection polarized Th1 cells produce IL-10 along to IFN-γ, in attempt to control immunopathology. The same has been described for monocytes. A recent article shows in the murine model of toxoplasmosis that Ly6C^hi^ monocytes entering the gastrointestinal tract responded to commensal ligands by adopting a regulatory phenotype. For instance inflammatory monocytes became capable to control parasite burden while limiting collateral damage to tissue [53]. Indeed, plasma levels of IL-10 are lower with increased disease severity during *P. vivax* infection [42]. Thus, the expression of this counter regulatory cytokine may also represent an important role of CD14^+^CD16^+^ monocytes in preventing immunopathology during *P. vivax* malaria.

Different adhesion molecules, including CD36, ICAM-1, VCAM-1 and PECAM-1 have been described as important receptors that bind *P. falciparum* infected red blood cells and influence the outcome of disease [54], [55]. *P. falciparum*-infected erythrocytes are able to tether and roll on CD36, ICAM-1, P-selectin, and VCAM-1 in a shear-dependent fashion. In addition, CD36 has an important role in phagocytosis of *P. falciparum* infected cells [56], [57], [58]. On the other hand, ICAM-1, but not CD36, was implicated in the cythoadhesion of *P. vivax* to endothelium cells [22]. We found that CD14^+^CD16^+^CCR2^+^ inflammatory monocytes from *P. vivax* malaria patients express the highest levels of ICAM-1, PECAM-1, and LFA-1. Although these adhesion molecules were originally identified as endothelium receptors for parasitized red blood cells, their expression on monocytes may favor biding and uptake of *P. vivax* infected reticulocytes. Indeed, our results indicate that the phagocytic activity of different monocyte subsets positively correlated with the expression of ICAM-1, PECAM-1 and LFA-1 and blockade of each of these adhesion molecules efficiently inhibited phagocytosis of Pv-Ret.

It is noteworthy that CD14^+^CD16^+^ monocytes have also been reported to expand in a group of *P. falciparum*-infected patients, and total monocytes from these patients were able to better control parasite growth *in vitro*, through antibody dependent cellular inhibition [59], [60]. CD16, a Fcγ receptor, has a high affinity for IgG1 and IgG3 [61] and therefore may be involved in phagocytosis of *Plasmodium* infected red blood cells. In contrast, experiments performed with *Staphylococcus aureus* or *Echerichia coli* showed that CD14^+^ monocytes have higher phagocytic activity than CD14^lo^ monocytes. However no differences were observed between CD14^+^CD16^+^ and CD14^+^CD16^−^ monocytes [62]. In our system, the expression of CD16 was not the only molecule involved in phagocytosis since the ability of Pv-Ret internalization was different between CD14^+^ and CD14^lo^, both expressing CD16. The expression of adhesion molecules ICAM-1, PECAM-1 and LFA-1, though, appeared to play an important role in parasite internalization by inflammatory monocytes. In vitro studies have shown that hemozoin interferes in the upregulation of MHC class II and CD54 on monocytes after IFN-γ stimulation [63], altering also their differentiation and maturation in dendritic cells [64] and antigen presentation [65]. Despite hemozoin is known to impair the ability of monocytes to repeat phagocytosis [66], no pigment was found when PBMC were evaluated in our study. Moreover, impairment in phagocytosis was not observed and the monocytes still produced ROS in response to PMA, in oppose to monocytes previously exposed to parasitized red blood cells [63]. We believe that phagocytosis of *P. vivax*-infected reticulocytes by monocytes are taking place in the spleen, where bona fide undifferentiated monocytes reside in equivalent numbers in circulation [67].

Besides their activated phenotype, CD14^+^ monocytes displayed an activated morphology with larger mitochondrias. Interestingly, the CD14^+^CD16^+^ subset showed higher mitochondrial activity than the other monocyte subpopulations. Malaria infection triggers production of high levels of total ROS. Despite similar levels of total ROS are produced by different monocyte subsets, higher frequencies and levels of intracellular ROS, and higher expression of p47phox and p67phox were found in CD14^+^CD16^+^ cells compared to CD14^+^CD16^−^ and CD14^lo^CD16^+^ monocytes. In addition, staining with MitoSox reveals that CD14^+^CD16^+^ monocytes produce higher levels of mitochondrial ROS. ROS are important effector free radicals involved in *Plasmodium* killing [47], [68]. Indeed, either blockade of mitochondrial complex I or blockage of NADPH oxidases, both responsible for ROS generation, efficiently reduces ROS levels in PBMC and CD14^+^CD16^+^ monocytes from malaria patients. Despite mitochondrial ROS have been regarded as byproducts of oxidative respiration, studies have indicated that mitochondria are recruited to vacuoles containing pathogens through an active process mediated by immune signaling [69], [70], [71]. Moreover, West and coworkers showed that the reduction of macrophage mitochondrial ROS results in defective bacterial killing [71].

Similarly to ROS production, higher frequencies of cells producing TNF-α, a cytokine known to trigger the respiratory burst, were found among CD14^+^CD16^+^ monocytes exposed to Pv-Ret. In a different context, patients experiencing severe malarial anemia triggered by *P. falciparum* display increased numbers of circulating monocytes, with significant augment in the numbers of TNF-α-producing CD14^+^CD16^+^ monocytes [72]. It is still unclear what is the cause of anemia during malaria. Despite recent reports have shown evidence demonstrating that *P. vivax* malaria may be associated with higher frequency and more severe anemia than *P. falciparum*, most of these studies were performed with children already suffering from malnutrition and hospitalized subject [73], [74], [75]. Only a small proportion of the *P. vivax*-infected patients analyzed in this study presented mild anemia (Table S1) and their reticulocyte counts are according to the reference values [76]. In fact, *P. vivax* infection is responsible for very low frequencies of infected red blood cells. We believe that during uncomplicated vivax malaria, phagocytosis of reticulocytes by monocytes and the production of inflammatory mediators will preferentially help in the parasite control.

It is important to mention that *P. vivax*-infected patients display elevated levels of hepatic biomarkers such as aspartate (AST), alanine (ALT) aminotransferase and bilirubin. And despite no correlation was found between monocyte activation markers and most of the laboratory parameters, such as hematocrit and platelet counts, higher levels of AST correlated with higher expression of CCR2 and VCAM-1 on both CD14^+^CD16^−^ and CD14^+^CD16^+^ monocytes and ICAM-1 on CD14^+^CD16^−^ monocytes (Figure S3). These data indicate that in the attempt of controlling parasitemia, monocytes might cause inflammatory damage.

Finally, we observed that CD14^lo^CD16^+^ patrolling monocytes from *P. vivax* malaria patients did not express CCR2 but expressed high levels of LFA-1 and CX3CR1. The latter two receptors appear to be responsible for the ability of monocytes to patrol blood vessels *in vivo* [18]. Consistent with this hypothesis, CD16^+^ monocytes display enhanced capacity to adhere to endothelial cells *in vitro*. This ability is partially dependent on fractalkine, the CX3CR1 ligand and CD11a, the α chain of LFA-1 [77]. Importantly, patrolling monocytes have been shown to express high levels of TLR8 and TLR9 [18], and may also play an important role on cytokine production in response to parasite RNA and DNA. Thus, patrolling monocytes may also contribute to the early inflammatory response observed during *P. vivax* infection.

In conclusion, our findings support the concept that highly activated monocytes are characteristic of acute malaria. *P. vivax*-infection leads to cytokine production by classical and inflammatory monocytes, and this response is likely to be largely responsible for many of the signs and symptoms observed in malaria sepsis. Importantly, we demonstrate for the first time that CD14^+^CD16^+^ monocytes in malaria patients exhibited greater phagocytic activity and produced higher levels of intracellular TNF-α and reactive oxygen species, indicating their important role in parasite control and host resistance to infection. Further delineation of the differential roles of monocyte subsets in *P. vivax* malaria could lead to identification of specific targets for therapeutic intervention in this extremely important but highly neglected parasitic disease.

## Supporting Information

Figure S1 **Size and granularity of human monocyte subsets.** Monocyte subsets are show in a forward scatter area (FSC-A) versus forward scatter height (FSC-H) dot plot. CD14^+^CD16^−^ cells are shown in green, CD14^+^CD16^+^ cells in red and CD14^l^°CD16^+^ in blue. Other cells from PBMC are shown in grey.
(EPS)

Figure S2 **Apoptosis of monocyte subsets after phagocytosis.** (A) Proportions of monocyte subsets and (B) frequencies of Annexin-V expressing CD14^+^CD16^−^ (green symbols), CD14^+^CD16^+^ (red symbols) and CD14^l^°CD16^+^ (blue symbols) monocytes in from *P. vivax*-infected patients. Cells were culture for 4 hours with *P. vivax*-infected reticulocytes (circles), 12 hours in medium alone (squares) or with *P. vivax*-infected reticulocytes (triangles). Symbols represent individual subject. * *p*<0.05.
(EPS)

Figure S3 **Levels of aspartate aminotransferase (AST) correlate with immunological parameters expressed by monocytes from** ***P. vivax*** **-infected patients.** (A) Levels of AST before (BT) and after (AT) treatment. Correlation between AST levels and CCR2, CD54, CD106 expression by CD14^+^CD16^−^ (B) or CD14^+^CD16^+^ (C) monocytes. The results are expressed as scattering of individual values. Circles indicate individual patients (n = 27). Spearman's rank correlation (r) and p values are shown in the graphs.
(EPS)

Figure S4 **Production of reactive oxygen species during vivax malaria.** Peripheral blood mononuclear cells and purified monocyte subsets from *P. vivax*-infected subjects were assessed for ROS production. (A) The kinetics of ROS production by PBMC from *P. vivax*-infected patients evaluated before (BT) and after treatment (AT) when cells were cultured in medium alone (left panel) or PMA (right panel) in the presence of luminol measured by chemiluminescence using Synergy H4 (BioTek) microplate reader (n = 5). (B) The kinetics of ROS production by sorted CD14^+^CD16^−^ (green circles), CD14^+^CD16^+^ (red circles) and CD14^l^°CD16^+^ (blue circles) from acutely *P. vivax*-infected patients cultured in medium alone (top panel), erythrocytes (RBC, middle panel) or *P. vivax*-infected reticulocytes (Pv-Ret, bottom panel) in the presence of carboxy-H_2_DCFDA measured by fluorescence using SpectraMax M5 microplate reader (n = 4). (C) The kinetics of ROS production by PBMC from acutely *P. vivax*-infected patients cultured in medium alone (top panel) or PMA (bottom panel), in the presence of luminol, in the absence or in the presence of inhibitors of ROS production; rotenone and DPI. Bars represent mean values and standard error. Results were pooled from 4 independent experiments. Luminol was measured by chemiluminescence (n = 5). Statistical analysis were done comparing different treatments over time, **0.05>*p*>0.01. (D) Mean fluorescence intensity (MFI) of ROS production was assessed by flow cytometry in CD14^+^CD16^+^ monocytes, among PBMC, from *P. vivax*-infected patients after culture with *P. vivax*-infected reticulocytes in the absence or in the presence of rotenone and DPI. Percentage of inhibition was calculated considering as 100% the culture in the absence of inhibitors (n = 4). Symbols represent individual subject. Results are representative of 2 experiments.
(EPS)

Table S1 **Study population.** Laboratory and clinical records of *Plasmodium vivax*-infected patients.
(DOCX)

Text S1 **Supporting methods for apoptosis assay and ROS detection.**
(DOCX)
