# Supplementary material for: The CD14+CD16+ Inflammatory Monocyte Subset Displays Increased Mitochondrial Activity and Effector Function During Acute Plasmodium vivax Malaria
Source: PLoS Pathog. 2014 Sep 18;10(9):e1004393. doi: 10.1371/journal.ppat.1004393 (PMC4169496; doi:10.1371/journal.ppat.1004393)
Supplement: Table S1 — Study population. Laboratory and clinical records of Plasmodium vivax-infected patients. (DOCX) [file ppat.1004393.s005.docx]

| Table S1. Study Population | | | | | |  | | |  | | | | | | | |  | | |
| --- | --- | --- | --- | --- | --- | --- | --- | --- | --- | --- | --- | --- | --- | --- | --- | --- | --- | --- | --- |
| **Patient ID** | **Gender** | **Age** | **# Malaria Episodes** | | | **Parasitemia. parasites/μl of blood** | | | **Hematological Records** | | | | | | | | **Symptoms** | | |
|  |  |  | 1st | <5 | ≥5 | *≤ 500* | *500 - 10000* | *> 10000* | *Hemoglobin g/dL.* | *Hematocrit %* | *Red Blood Cells x10^6^/mm^3^* | *White Blood Cells x10^3^/mm^3^* | *Platelets x 10^3^/mm^3^* | *Creatinine mg/dL* | *AST U/L* | *ALT U/L* | Myalgia | Headache | Chills |
| P23 BT* | Male | 36 |  | X |  | X |  |  | 15.5 | 44.4 | 4.88 | 4.2 | 89 | 1 | 15 | 35 | Yes | Yes | Yes |
| P23 AT** | Male | 36 |  |  |  | Not detected*** | | | ND**** | ND | ND | ND | ND | ND | ND | ND | No | Yes | ND |
| P24 BT | Female | 25 |  |  | X |  | X |  | 13.1 | 38.6 | 4.88 | 6.8 | 138 | 0.83 | 21 | 33 | Yes | Yes | Yes |
| P24 AT | Female | 25 |  |  |  | Not detected | | | 14.2 | 43.1 | 5.35 | 6.4 | 305 | 0.5 | 16 | 20 | No | No | No |
| P25 BT | Male | 39 |  |  | X | X |  |  | 14.2 | 41.2 | 4.66 | 4.6 | 96 | 1.1 | 24 | 32 | Yes | Yes | Yes |
| P25 AT | Male | 39 |  |  |  | Not detected | | | 13.7 | 40.9 | 4.71 | 4.0 | 188 | 0.48 | 12 | 18 | No | No | No |
| P26 BT | Male | 41 |  |  | X |  | X |  | 14.2 | 41.6 | 4.7 | 6.1 | 112 | 1 | 31 | 20 | Yes | No | Yes |
| P26 AT | Male | 41 |  |  |  | Not detected | | | 14 | 47.7 | 4.82 | 5.0 | 121 | 0.55 | 29 | 20 | No | No | No |
| P27 BT | Male | 19 | X |  |  |  |  | X | 12.1 | 35.0 | 4.4 | 9.5 | 94 | 0.86 | 44 | 37 | Yes | Yes | Yes |
| P27 AT | Male | 19 |  |  |  | Not detected | | | 14.8 | 44.2 | 5.28 | 6.1 | 135 | 0.5 | 22 | 20 | No | No | No |
| P28 BT | Female | 41 |  |  | X | X |  |  | 13.8 | 40.2 | 4.45 | 7.7 | 120 | 0.7 | 12 | 16 | Yes | Yes | Yes |
| P28 AT | Female | 41 |  |  |  | Not detected | | | 13.3 | 40.9 | 4.53 | 7.0 | 171 | 0.9 | 20 | 15 | No | No | No |
| P29 BT | Male | 25 |  | X |  |  |  | X | 13.8 | 41.8 | 5.25 | 6.3 | 74 | 1.3 | 22 | 29 | Yes | Yes | Yes |
| P29 AT | Male | 25 |  |  |  | Not detected | | | ND | ND | ND | ND | ND | ND | ND | ND | ND | ND | ND |
| P30 BT | Male | 38 | X |  |  |  | X |  | 12.4 | 38.4 | 4.16 | 3.3 | 47 | 0.92 | 25 | 21 | Yes | Yes | Yes |
| P30 AT | Male | 38 |  |  |  | Not detected | | | ND | ND | ND | ND | ND | ND | ND | ND | ND | ND | ND |
| P31 BT | Male | 28 |  |  | X |  | X |  | 14.4 | 43.7 | 4.98 | 4.7 | 41 | 0.3 | 39 | 31 | Yes | No | Yes |
| P31 AT | Male | 28 |  |  |  | Not detected | | | 15.5 | 46.9 | 5.54 | 6.5 | 177 | 0.49 | 40 | 38 | No | No | No |
| P32 BT | Male | 28 |  |  | X | X |  |  | 12.8 | 37.1 | 4.38 | 7.6 | 150 | 0.7 | 43 | 69 | Yes | Yes | No |
| P32 AT | Male | 28 |  |  |  | Not detected | | | ND | ND | ND | ND | ND | ND | ND | ND | ND | ND | ND |
| P33 BT | Male | 34 |  |  | X | X |  |  | 13.1 | 37.6 | 4.31 | 5.1 | 151 | 0.6 | 11 | 15 | Yes | Yes | Yes |
| P33 AT | Male | 34 |  |  |  | Not detected | | | 13.6 | 39.1 | 4.51 | 8.0 | 249 | 0.9 | 16 | 15 | No | No | No |
| P34 BT | Male | 24 |  | X |  | X |  |  | 16.3 | 47.3 | 5.29 | 6.9 | 107 | 1.1 | 26 | 26 | Yes | No | No |
| P34 AT | Male | 24 |  |  |  | Not detected | | | ND | ND | ND | ND | ND | ND | ND | ND | ND | ND | ND |
| P35 BT | Male | 27 | X |  |  | X |  |  | 13.2 | 39.7 | 4.83 | 4.8 | 124 | 0.81 | 91 | 45 | Yes | Yes | Yes |
| P35 AT | Male | 27 |  |  |  | Not detected | | | 13.2 | 39.7 | 4.83 | 4.8 | 124 | 0.81 | 91 | 45 | ND | ND | ND |
| P36 BT | Male | 44 |  | X |  |  | X |  | 14.3 | 42 | 4.82 | 6.9 | - | 0.07 | 47 | 20 | Yes | Yes | No |
| P36 AT | Male | 44 |  |  |  | Not detected | | | 15.8 | 45.3 | 5.14 | 5.5 | 189 | 0.7 | 13 | 12 | No | No | No |
| P37 BT | Male | 38 | X |  |  |  | X |  | 13.6 | 41.4 | 5.24 | 4.0 | 52 | 0.83 | 85 | 68 | Yes | Yes | Yes |
| P37 AT | Male | 38 |  |  |  | Not detected | | | 15.9 | 47.7 | 5.82 | 6.9 | 238 | 0.4 | 14 | 15 | No | No | No |
| P38 BT | Male | 45 |  | X |  |  | X |  | 13.3 | 41.9 | 4.42 | 5.1 | 65 | 1 | 64 | 35 | Yes | Yes | Yes |
| P38 AT | Male | 45 |  |  |  | Not detected | | | 17.4 | 50.6 | 5.35 | 4.9 | 195 | 0.9 | 23 | 42 | No | No | No |
| P39 BT | Male | 37 | X |  |  | X |  |  | 13.6 | 39.1 | 4.84 | 2.5 | 30 | 1.09 | 172 | 178 | Yes | Yes | ND |
| P39 AT | Male | 37 |  |  |  | Not detected | | | 14.5 | 43.1 | 5.2 | 4.7 | 94 | 1.09 | 25 | 21 | No | No | No |
| P40 BT | Male | 42 |  | X |  |  | X |  | 14.8 | 44.6 | 5.36 | 5.3 | 148 | 0.9 | 32 | 61 | Yes | Yes | Yes |
| P40 AT | Male | 42 |  |  |  | Not detected | | | 14.4 | 44.8 | 5.41 | 6.5 | 261 | 0.6 | 53 | 78 | No | No | No |
| P41 BT | Male | 18 |  |  | X | X |  |  | 13.2 | 40.4 | 5.09 | 5.5 | 53 | 1 | 16 | 17 | Yes | Yes | Yes |
| P41 AT | Male | 18 |  |  |  | Not detected | | | ND | ND | ND | ND | ND | ND | ND | ND | ND | ND | ND |
| P42 BT | Male | 46 |  |  |  | X |  |  | 11.1 | 32.2 | 3.6 | 2.0 | 44 | 0.73 | 22 | 31 | ND | ND | ND |
| P42 AT | Male | 46 |  |  |  | Not detected | | | 14.7 | 42.6 | 4.63 | 3.8 | 101 | 0.9 | 24 | 22 | No | No | No |
| P43 BT | Male | - |  | X |  |  | X |  | 6.7 | 23.1 | 2.62 | 3.4 | 220 | 0.8 | 20 | 36 | Yes | Yes | Yes |
| P43 AT | Male | - |  |  |  | Not detected | | | 13.2 | 41 | 4.59 | 5.8 | 161 | 0.7 | 23 | 19 | No | No | No |
| P44 BT | Female | - |  | X |  |  | X |  | 11.7 | 35.9 | 4.25 | 6.6 | 75 | 0.7 | 29 | 44 | Yes | Yes | Yes |
| P44 AT | Female | - |  |  |  | Not detected | | | 11.1 | 34.8 | 4.03 | 7.3 | 305 | 0.7 | 30 | 27 | No | No | No |
| P45 BT | Male | - |  | X |  | X |  |  | 14.1 | 42.6 | 5.36 | 5.2 | 153 | 0.9 | 32 | 30 | Yes | No | Yes |
| P45 AT | Male | - |  |  |  | Not detected | | | 14.8 | 46.2 | 5.78 | 6.7 | 242 | 0.92 | 45 | 40 | No | No | No |
| P46 BT | Female | - | X |  |  |  |  | X | 11.8 | 35.5 | 3.93 | 3.3 | 29 | 0.9 | 58 | 46 | Yes | Yes | Yes |
| P46 AT | Female | - |  |  |  | Not detected | | | 12.7 | 39.8 | 4.18 | 6.9 | 193 | 0.9 | 46 | 43 | No | No | No |
| P47 BT | Male | - |  | X |  |  |  | X | 13.2 | 39.9 | 4.35 | 5.5 | 112 | 0.7 | 31 | 30 | Yes | No | No |
| P47 AT | Male | - |  |  |  | Not detected | | | ND | ND | ND | ND | ND | ND | ND | ND | ND | ND | ND |
| P48 BT | Male | - |  |  | X |  |  | X | 14.2 | 43.1 | 4.89 | 4.1 | 179 | 0.7 | 38 | 51 | Yes | Yes | Yes |
| P48 AT | Male | - |  |  |  | Not detected | | | 11.9 | 38.5 | 4.4 | 5.7 | 201 | 1 | 26 | 23 | No | No | No |
| P49 BT | Male | - |  | X |  |  | X |  | 14.6 | 42.3 | 5.03 | 4.1 | 132 | 0.8 | 89 | 183 | Yes | Yes | Yes |
| P49 AT | Male | - |  |  |  | Not detected | | | 13.6 | 40.2 | 4.88 | 6.7 | 228 | 0.9 | 18 | 24 | No | No | No |
| P50 BT | Male | - |  | X |  |  | X |  | 17.7 | 51.3 | 6.19 | 4.2 | 61 | 0.8 | 23 | 24 | Yes | Yes | Yes |
| P50 AT | Male | - |  |  |  | Not detected | | | 14.8 | 43.8 | 5.37 | 4.7 | 142 | 0.8 | 17 | 15 | No | No | No |
| P52 BT | Male | - |  | X |  | X |  |  | 14.6 | 43.7 | 4.86 | 6.8 | 235 | 1 | 36 | 29 | Yes | Yes | Yes |
| P52 AT | Male | - |  |  |  | Not detected | | | ND | ND | ND | ND | ND | ND | ND | ND | ND | ND | ND |

*Before treatment

**After treatment

***Parasitemia not detected by blood smear and PCR

****Not determined
