# Supplementary material for: The CD14+CD16+ Inflammatory Monocyte Subset Displays Increased Mitochondrial Activity and Effector Function During Acute Plasmodium vivax Malaria
Source: PLoS Pathog. 2014 Sep 18;10(9):e1004393. doi: 10.1371/journal.ppat.1004393 (PMC4169496; doi:10.1371/journal.ppat.1004393)
Supplement: Text S1 — Supporting methods for apoptosis assay and ROS detection. (DOCX) [file ppat.1004393.s006.docx]

**Text S1**

**Supplemental Material**

**Supplemental Methods**

**Apoptosis assay.** Apoptosis was assessed by incubating PBMC in suspension in 5 mL polystyrene tubes at 37°C in complete RPMI 1640 supplemented 2 µM glutamine, 10 mM HEPES, and 50 µM 2-ME for 4 and 12 hours with *P. vivax*-Ret (0.5 Pv-Ret/PBMC) in the presence of 10% immune serum (*P. vivax*-infected patient). PBMC were then washed with FACS buffer (PBS 2%SBF), incubated for 15 minutes with monoclonal antibodies in FACS buffer to stain the monocyte subsets. The antibody panel is described in the manuscript. Cell suspensions were washed with cold PBS, and stained with Annexin-V in binding buffer, using a commercially available kit. At least 100,000-gated events were acquired for analysis using a digital flow cytometer. The frequencies of each subpopulation undergoing apoptosis were determined by flow cytometry. Data were analyzed using FlowJo Version X 10.0.7.

**ROS detection.** Luminol (Sigma) and carboxy-H_2_DCFD were used to detect total ROS production. In the experiments in which ROS were measured in PBMC from malaria patients, before and after treatment, cells were separated in the endemic area, frozen and transported to the Laboratory of Immunopatology in Belo Horizonte (Figure S4A). Blood samples were otherwise collected in the endemic area of malaria and shipped overnight at room temperature to the laboratory in Belo Horizonte where PBMC were immediately separated as described and monocyte subpopulations purified by FACS at 4˚C. After purification cells were kept on ice for further analysis. Cultures were prepared with 2.5x10^5^ PBMC or 1.25x10^5^ FACS-sorted monocyte subsets (CD14^+^CD16^-^, CD14^+^CD16^+^, CD14^lo^CD16^+^ cells) in HBSS alone, erythrocytes, Pv-Ret (1 Pv-Ret/PBMC) or PMA (10ng/mL) and ionomycin (500ng/mL). Cell suspensions were incubated at 37°C for 1:30 hours while measurement of ROS by luminol (relative light unit, RLU) and by H_2_DCFDA (relative fluorescence unit, RFU) was performed every 10 min. In some experiments cells were pre-incubated for 30 min with 10 μM rotenone and 5 μM DPI (diphenylene iodonium) to block the mitochondria complex I and NADPH oxidase plus nitric oxide synthases, respectively. H_2_DCFDA was analyzed with SpectraMax M5 and luminol with Synergy H4 (BioTek) microplate readers.

Additionally, ROS production was also assessed at the single cell level. PBMC were washed twice with PBS and pre-incubated with rotenone and DPI. After 30 min medium or Pv-Ret (0.5 Pv-Ret/PBMC) and pre-warmed 25 µM H_2_DCFDA were added to the cultures for 3 hours at 37°C. Cells were then washed, incubated at room temperature for 20 min with anti-CD14 and anti-CD16. After staining, plates were kept on ice for 15 min and cells were harvested with ice-cold PBS containing 2.5mM EDTA. Cells were acquired by flow cytometry and data were analyzed using FlowJo Version 9.3.2.
